# Artificial intelligence in plastic surgery, where do we stand?

**DOI:** 10.1016/j.jpra.2024.09.003

**Published:** 2024-09-14

**Authors:** Omar Kiwan, Mohammed Al-Kalbani, Arash Rafie, Yasser Hijazi

**Affiliations:** a Plastic and Reconstructive Department, Lancashire Teaching Hospitals NHS Foundation, United Kingdom; b Faculty of Biology, Medicine and Health, University of Manchester, United Kingdom

**Keywords:** Artificial intelligence, Plastic surgery, Machine learning, Aesthetic surgery

## Abstract

Since the pandemic, artificial intelligence (AI) has been integrated into several fields and everyday life as well. Healthcare is not an exception. Plastic surgery is a key focus area of this technological revolution, with hundreds of studies and reviews already published on the use of AI in plastics. This review summarizes the entirety of the available literature from 2020 to provide a comprehensive overview on AI innovation in plastic surgery.

A systematic literature review (following the PRISMA guidelines) of all studies and papers that examined the application of AI in plastic surgery was carried out using Medline, Cochrane, Embase, and Google Scholar.

Outcomes of interest included the growing role of AI in clinical consultations, diagnosing potentials, surgical planning, intraoperative, and post-operative uses. Ninety-six studies were included in this review; six examined the role of AI in consultations, fifteen used AI in diagnoses and assessments, seventeen involved AI in surgical planning, fifteen reported on AI use in post-operative predictions and management, and nine involved administrations and documentation.

This comprehensive review of available literature found AI to be capable of transforming care throughout the entire patient journey. Certain challenges and concerns persist, but a collaborative effort can solve these issues to bring about a new era of medicine, where AI aids doctors in the pursuit of optimal patient care.

## Introduction

Since the pandemic, artificial intelligence (AI) has been integrated into several fields and everyday life as well. Healthcare is not an exception. Organizations such as the United Kingdom's National Health Service have invested large sums into Healthcare AI development. It is expected that the NHS’ 21-million-pound investment on AI tools will result in economic gains of 235 million pounds over the next 5 years.^1^ AI has the potential to provide a creative solution to significant issues in the NHS caused by the lack of funding,^2^ such as the staffing crisis.^3^

Plastic surgery is a key focus area of this technological revolution, with hundreds of studies and reviews already published on AI use in the field. Integrating technology has long been a focus of plastic surgery. Robotic surgery, for example, is being established in plastics, with several studies demonstrating the improved safety and outcomes.^4^^,^^5^ The scope and potential benefits of AI are much more widespread, as it could impact the entire patient journey from the initial assessment to post-op monitoring. This review looked into the growing role of AI in clinical consultation, diagnostic potentials, surgical planning, intraoperative aid, and post-operative monitoring in the field of plastic surgery.

## Methods

A systematic literature review (following the PRISMA guidelines) of all studies and papers that examined AI in plastic surgery was performed using Medline, Cochrane, Embase, and Google Scholar. Outcomes of interest included the growing role of AI in clinical consultations, diagnosing potentials, surgical planning, intraoperative, and post-operative uses.

## Results

**Figure 1:**
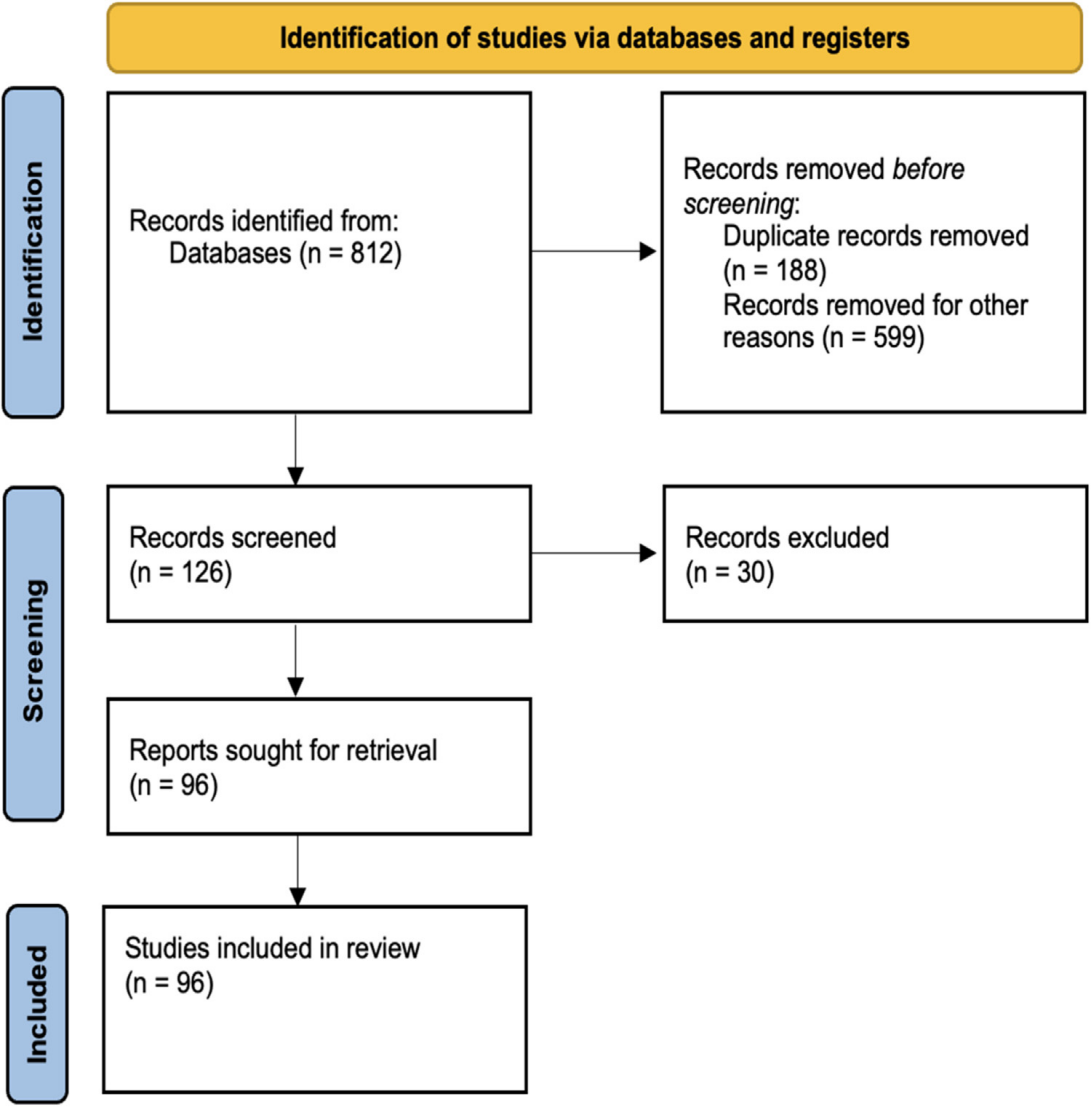
Flow-gram of study selection.

**Figure 2:**
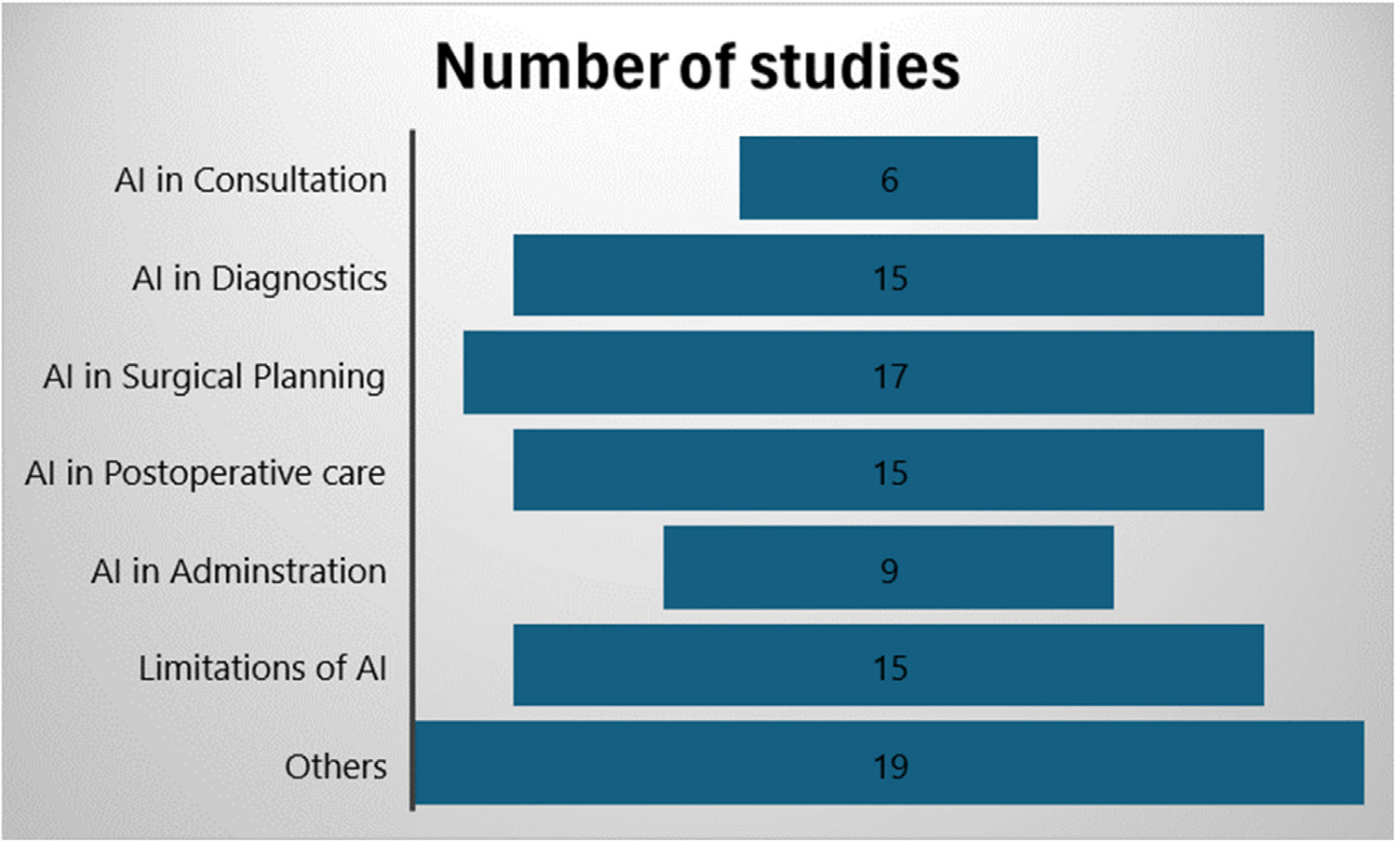
Categories of the assorted studies used. Studies categorized as “Other” include those in education, research, and imaging.

**Figure 3:**
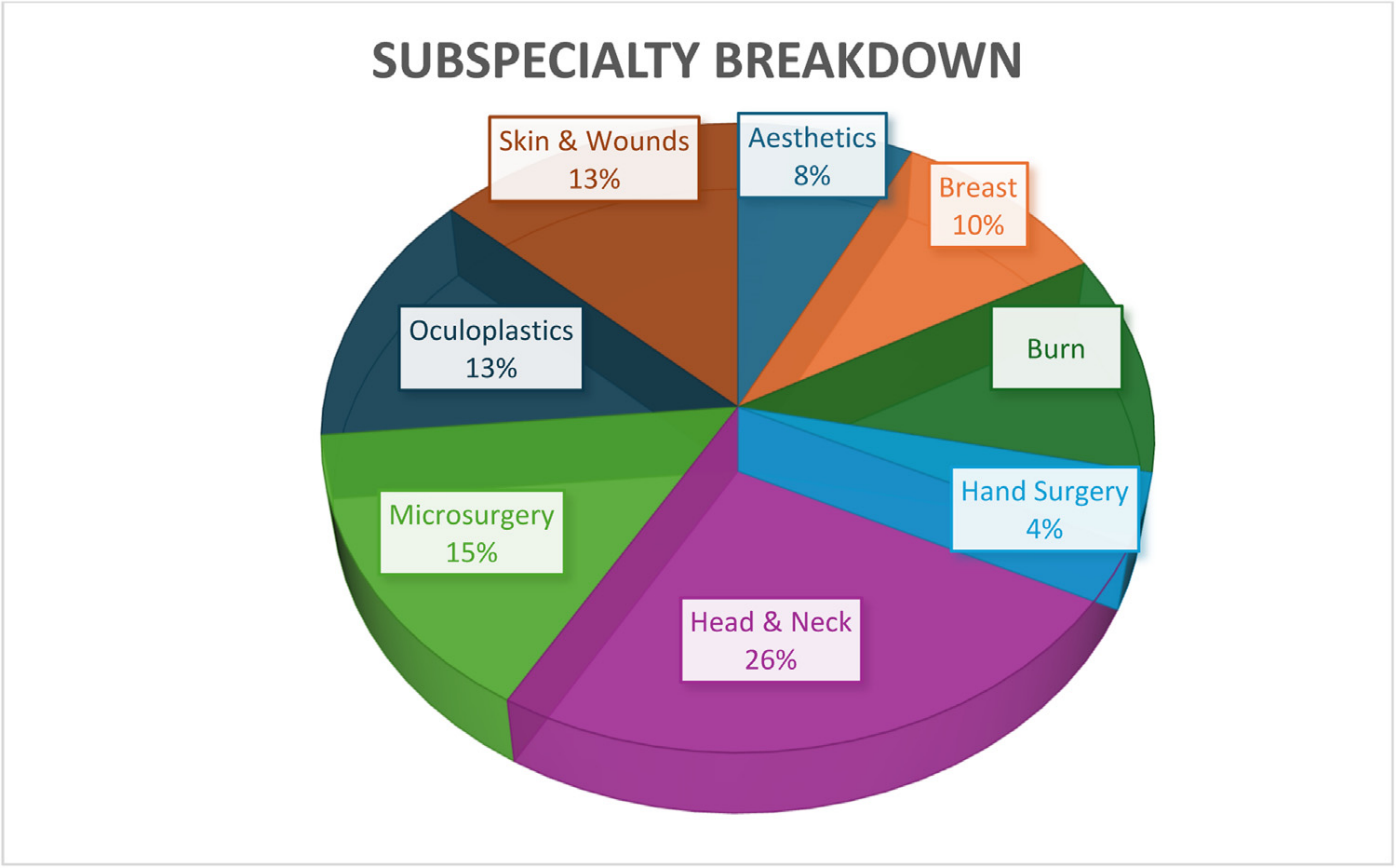
Subspecialty breakdown in different aspects of plastic surgery.

Following the identification of 812 studies that involved AI in surgery, 716 studies were excluded so that only those related to plastic surgery were included (Figure 1). Among the 96 remaining studies, six examined the role of AI in consultations, fifteen used AI in diagnoses and assessments, seventeen involved AI in surgical planning, fifteen checked AI in post-operative predictions and management, and nine involved administration and documentation (Figure 2). In addition, four were related to aesthetic surgery, 5 to breast surgery, six to burns, two to hand surgery, fourteen to head and neck, eight to microsurgery, seven to oculoplastics, and seven to skin and wounds (Figure 3). The reasons for exclusion were that the study is not relevant, is a duplicate, is an opinion, has no clinical role, is an animal study, or that the translation could not be found.

### AI in clinical consultations

Consultations are essential in formulating the right differentials and management plan for a patient. They also help build patient rapport and trust in the medical team. Having AI take over consultations is ambitious and difficult to accept. When it comes to knowledge, the popular AI chatbot chat generative pre-trained transformer (ChatGPT) has been shown to perform on a similar level to a first-year plastics trainee on the in-service exam, achieving a score in the 49th percentile.^6^ It is likely that AI trained in plastic surgery would achieve a much higher score. A study using ChatGPT for blepharoptosis consultations found that it provided better information than experienced plastic surgeons.^7^ Furthermore, ChatGPT was found to provide better responses to microsurgery-related questions than the website of the American society of reconstructive microsurgery, according to plastic surgeons and non-medical individuals.^8^

Two studies demonstrated the ability of AI to provide objective feedback,^9^^,^^10^ and one study found that ChatGPT can demonstrate empathy^11^ meaning with the right knowledge, it might provide accurate diagnoses and management of various conditions. However, there are also examples of ChatGPT being poor at consultations; a study asking ChatGPT to gain informed consent for surgery found it to be inaccurate and not informative enough to gain consent.^12^

Overall, complete AI takeover in consultations is hard to perceive. It would need to pass an excruciating number of trials to be deemed safe and acceptable. A supportive role for AI in consultations is much more likely. AI has been shown to provide more accurate predictions of post-op morbidity and mortality than the widely used predictors, such as the American society of anesthesiologists (ASA) score, to patients, which augments the process of obtaining surgical consent.^13^

AI systems, such as data analysis expressions (DAX) copilot, have been shown to automatically and efficiently document patient encounters.^14^ DAX copilot has been shown to improve clinician and patient satisfaction,^15^ likely because doctors can focus more on patient centered encounters without the need to look at a screen. It would also give doctors time to see more patients without needing to document the previous consultation first, making the outpatient service more efficient.

### Diagnostic potential of AI

Training AI to identify diagnoses can help speed up assessments of patients and allow doctors to produce a management plan while waiting for the test results. Some surgical specialties have begun using AI to assess patients. In colorectal surgery, AI is used with colonoscopies to detect polyps and adenomas. A meta-analysis of 48 studies found that AI use significantly increased the detection rates.^16^ In plastic surgery, studies have found AI to be capable of diagnosing skin cancers,^17^ lagophthalmos,^18^ frostbite,^19^ and velopharyngeal insufficiency.^20^ AI can diagnose these conditions using images, indicating that smartphone applications should be capable of diagnosis without the need for complex, expensive machinery. A prospective study developed an AI integrated smartphone app that was 87.5 % sensitive in automated diagnosis of positional plagiocephaly in infants.^21^ In addition, AI use in elastic-scattering spectroscopy has been shown to be highly sensitive in detecting different skin cancers, although the device's specificity is very low.^22^

Furthermore, AI can assess the severity of different conditions. In burn injuries, for instance, a deep learning software was developed to classify wounds and was found to do so more precisely than specialists.^23^ AI predicted mortality following a burn as accurately as the original and revised Baux score.^24^ A second study demonstrated that AI could predict numerous adverse effects in burn patients, such as the need for extended hospital stay and skin graft survival.^25^ A systematic review found that AI has the potential to transform burn care, and can advance diagnostic accuracy, management, and care efficiency.^26^

In the head and neck, an AI algorithm showed 100 % accuracy in grading facial palsy using the House and Brackmann Scale,^27^ and eight different AI networks were trialed to evaluate the severity of microtia and found all of them to be over 80 % accurate.^28^

Overall, AI has shown success in accurately diagnosing patients rapidly and without the need for any additional materials. However, most of these studies trained an AI to diagnose a specific condition. Practically, this would only be used to help confirm an already suspected diagnosis. AI needs to be trained to diagnose a wide range of conditions for it to diagnose independently.

### AI in surgical planning

Surgical planning takes time and requires accurate analyses of imaging, tests, and patient-specific characteristics and comorbidities. AI can segment bones and tissues on scans, allowing for the identification and detailed analysis of body landmarks needed for the surgery. Moreover, AI can segment ultrasound scans of adipose tissue prior to liposuction^29^ and the carpal tunnel to identify the median nerve.^30^ One study investigated the role of AI in deep inferior epigastric artery perforator (DIEP) flap execution.^31^ The anatomy of the DIEP is complex, and consequently, surgical planning using computed tomography (CT) angiograms is needed to select suitable perforators. AI could enhance the acquisition, processing, and interpretation of CT angiograms in mapping the course of the perforators, which would save valuable time. In addition to this, AI can identify surgically important anatomical landmarks, such as cleft lip and nasal deformity,^32^ from photographs and videos to further augment its potential role in surgical planning. A deep learning program automatically segmented the mandible more accurately than semi-automatic segmentation, which is the present clinical standard, and in a fraction of the time.^33^

In nerve histomorphometry analysis, AI was found to be more accurate and quicker than conventional methods.^34^ One study used the AI abilities to recognize facial expressions from a facial recognition software on iPad, to aid surgical reanimation planning.^35^ In burn injuries, the depth of the burn can be analyzed using AI by segmenting the burn area from the entire wound and using a pixel-to-pixel method to support excision planning.^36^ In oculoplastic, AI can also measure the marginal reflex distance1 accurately and rapidly to plan blepharoplasty.^37^

The use of three-dimensional (3D) printing is becoming more prevalent in plastic surgery. 3D printing has already been applied to the breast,^38^ ear,^39^ and nasal reconstruction^40^ and has improved surgical outcomes and satisfaction.^41^ 3D printing can produce patient-specific implants, based on CT and magnetic resonance imaging,^42^ to rehabilitate function and aesthetics.^43^ Currently, 3D printing is only used on a small scale due to its complexity. The process is time consuming and involves digital reconstruction, optimization, geometrical support, and quality control.^42^ AI can help streamline this process by assisting in image segmentation, modifying designs, and automatically generating optimized support structures to produce high quality prosthetics in a cost effective and efficient manner.^44^

### Intraoperative uses of AI

AI can play a productive role by providing accurate assessments of intraoperative situations. A study found that AI can recognize whether a wound bed is viable, to help determine when to stop excision.^45^ In fact, it also found that whenever a surgeon changed their mind following an AI input, they always changed from the incorrect decision to the correct one.^45^ AI can also provide solutions to certain concerns, such as the use of ChatGPT to correctly answer intraoperative questions on the DIEP flap procedure.^46^

### Post-operative AI uses

Numerous studies have found AI to accurately assess surgical outcomes. An AI integrated smartphone app could determine rhinoplasty status as accurately as consultants.^47^ AI can objectively measure years regained after facial rejuvenation surgery^10^ and assess intervention success from a patient's perspective in aesthetic surgery.^9^ AI is capable of gauging free flap perfusion and viability using photoplethysmography.^48^^,^^49^

Moreover, AI has been used to predict post-operative complications. It was found to have a high accuracy in predicting outcomes and complications of free flaps following head and neck tumor surgery.^50^ Machine learning can predict infection and explantation of breast implants in reconstruction, it identified 9 and 12 significant predictors, respectively, and had an accuracy of over 80 %.^51^ MySurgeryRisk is an AI program developed to predict complications such as acute kidney injury, sepsis, and venous thromboembolism. It had higher accuracy in predicting complications than physicians. Physician accuracy also significantly improved following AI use.^52^ The machine learning risk calculator, Predictive OpTimal Trees in Emergency Surgery Risk (POTTER), was developed using data from over 380,000 emergency surgery patients. POTTER outperformed the widely used scoring systems, including ASA, in predicting post-surgical morbidity and mortality.^13^ AI could also detect post-operative complications of breast augmentation that were previously unknown; larger preoperative bra-cup sizes and increased height were found to increase the risk of complications in a study involving over 1600 patients.^53^ ChatGPT could come up with the most likely problem in 20 out of 22 post-operative aesthetic surgery presentations.^54^ Following the development of any complications, AI was shown to consistently manage them appropriately.^55^

Following surgery, resources and personnel are needed to continuously monitor the patient. Smartphones are readily available and their use in patient monitoring has been successful in conditions such as multiple sclerosis^56^ and COVID-19.^57^ In plastic surgery, a free heart rate app can be used to monitor fasciocutaneous perforator flap perfusion using its pulse.^58^ Continuous AI-based vital monitoring can also help reduce the risk of post-operative complications by alerting staff as soon as any issues arise.^14^ However, few AI integrated applications have been used in plastic surgery. A deep learning integrated app could predict free flap congestion with 95 % accuracy, it also achieved 95 % sensitivity and specificity.^59^ Overall, AI-based monitoring shows early promise but requires more studies to determine its viability.

### Other uses of AI

AI can contribute to research in plastic surgery. It can assist researchers in projects such as systematic reviews^60^ and can automate critical appraisals,^61^ although the latter is limited to abstracts due to accessibility. The publicly available AI, ChatGPT can also draft research articles.^62^ However, it is also known to make up sources when writing,^63^ which is a cause for concern and hampers the integrity of research. The integration of AI into medical databases could be a solution for this in the long run.

The use of AI to streamline administrative work has also been trialed in plastic surgery. A hand and reconstructive microsurgery department used AI NUHS Russel-GPT for administration work. A survey found that the staff unanimously supported its use.^64^ AI integration into administrative work will provide the staff with more time to assess and support the patients.

## Limitations of AI

Despite the numerous advancements in AI, several ethical concerns must be tackled before it can safely be integrated into healthcare. In plastic surgery, data protection and privacy are the main concern according to a systematic review,^65^ as any AI integration would likely require providing confidential patient information to a third party that creates and regulates the software. AI can also worsen healthcare inequality and exhibit patient bias, as data for machine learning software would come from a specific population group, likely younger and better off individuals. This has been showcased in a systematic review involving 86 publications.^66^ The review also found that AI could shift care to a profit-based system.

In addition to bias against the patients, AI was also found to exhibit bias against doctors. A study analyzing three commonly used AI software reported that AI recommends far fewer female doctors than the national proportion.^67^ Moreover, AI has been found to under-skill and over-skill different surgeons at different rates. However, this can be mitigated by teaching the AI to provide visual explanations for its assessments.^68^ Protocols such as PROBAST are also being developed to assess for biases in AI.^69^ In addition, plastics staff are largely inexperienced with AI^70^ and may require comprehensive training prior to utilization.

## Ongoing AI projects in plastic surgery

This section provides an overview of the ongoing projects that could contribute to innovation in plastic surgery. A randomized controlled trial is expected to start this year on an AI virtual assistant app that can help support patients before their plastic surgery. The results will be compared to a call group, which will require phoning a call center to ask questions.^71^ Trials are ongoing for the development of a machine learning algorithm that can predict and prevent intraoperative hypotension.^72^ Smart monitoring devices are also being trialed forward^73^- and home-based^74^ monitoring. The latter could decrease the need for surgical follow-ups and hospital stay.

## Discussion

The integration of AI into plastic surgery heralds an exciting new era of patient care. Comprehensive literature analysis has shown its ability to improve outcomes in each step of the patient journey.

The use of AI in supporting doctors, owing to its ability to assess medical imaging accurately and rapidly and to predict outcomes have been key features of this review and are likely the way forward. Automatic dictation would help doctors dedicate more attention to patients in consultations. The ability of AI to segment scans and identify key landmarks can take preoperative planning to a new level. Its prediction of post-operative complications can improve surgical decision making and provide patients with the most accurate information while obtaining informed consent. These developments would expedite and improve management and mitigate the risk of human error, thus improving patient safety and reducing costs.

Despite the remarkable progress within AI developments in plastic surgery, several challenges remain. Moreover, concerns on data privacy and algorithmic bias must be addressed. A robust authentication and regulation process must also be developed to ensure patient safety.

AI has the potential to reshape plastic surgery in previously unimaginable ways. Further research, regulation, and work on upholding the ethical standards can allow plastic surgeons to harness the power of AI to provide new and high standards of patient care.

To conclude, the comprehensive review of available literature found AI to be capable of transforming care throughout the entire patient journey. Certain challenges and concerns persist, but a collaborative effort can help solve these issues to bring about a new era of medicine, where AI aids doctors in the pursuit of optimal patient care.

## Ethical approval

Not required.

## Author contributions

All authors have participated in the conception and design of the study, acquisition of data, analysis, and interpretation of data, and drafting the article.

## Conflict of interest

The authors have nothing to disclose.
